# A gene frequency model for QTL mapping using Bayesian inference

**DOI:** 10.1186/1297-9686-42-21

**Published:** 2010-06-11

**Authors:** Wei He, Rohan L Fernando, Jack CM Dekkers, Helene Gilbert

**Affiliations:** 1 Department of Animal Science, Iowa State University, Ames, IA, USA; 2 Center for Integrated Animal Genomics, Ames, IA, USA; 3 INRA, UMR 1313 Génétique Animale et Biologie Intégrative, F-78352 Jouy-en-Josas, France

## Abstract

**Background:**

Information for mapping of quantitative trait loci (QTL) comes from two sources: linkage disequilibrium (non-random association of allele states) and cosegregation (non-random association of allele origin). Information from LD can be captured by modeling conditional means and variances at the QTL given marker information. Similarly, information from cosegregation can be captured by modeling conditional covariances. Here, we consider a Bayesian model based on gene frequency (BGF) where both conditional means and variances are modeled as a function of the conditional gene frequencies at the QTL. The parameters in this model include these gene frequencies, additive effect of the QTL, its location, and the residual variance. Bayesian methodology was used to estimate these parameters. The priors used were: logit-normal for gene frequencies, normal for the additive effect, uniform for location, and inverse chi-square for the residual variance. Computer simulation was used to compare the power to detect and accuracy to map QTL by this method with those from least squares analysis using a regression model (LSR).

**Results:**

To simplify the analysis, data from unrelated individuals in a purebred population were simulated, where only LD information contributes to map the QTL. LD was simulated in a chromosomal segment of 1 cM with one QTL by random mating in a population of size 500 for 1000 generations and in a population of size 100 for 50 generations. The comparison was studied under a range of conditions, which included SNP density of 0.1, 0.05 or 0.02 cM, sample size of 500 or 1000, and phenotypic variance explained by QTL of 2 or 5%. Both 1 and 2-SNP models were considered. Power to detect the QTL for the BGF, ranged from 0.4 to 0.99, and close or equal to the power of the regression using least squares (LSR). Precision to map QTL position of BGF, quantified by the mean absolute error, ranged from 0.11 to 0.21 cM for BGF, and was better than the precision of LSR, which ranged from 0.12 to 0.25 cM.

**Conclusions:**

In conclusion given a high SNP density, the gene frequency model can be used to map QTL with considerable accuracy even within a 1 cM region.

## Background

Molecular information is currently being used for mapping quantitative trait loci (QTL) and for genetic evaluation. This information usually consists of molecular genotypes at polymorphic loci. These loci can be broadly classified into two types: I) those that have a direct effect on the trait, and II) those that do not have a direct effect on the trait but are linked to a trait locus (markers). Loci of type II can be further classified into two types: IIa) loci that are in linkage disequilibrium with the trait locus across the population (LD markers), and IIb) loci that are in linkage equilibrium with the trait locus (LE markers) [1]. In outbred populations, until recently, marker analyses were primarily based on LE markers [2-6]. LE markers do not provide information to model the mean at linked QTL, but they do provide information to model covariances at the linked QTL. These covariances can be written in terms of the conditional IBD probabilities at a linked QTL [2,5,6] and provide information to map QTL and for genetic evaluation using markers. This cosegregation (CS) information comes from the non-random association of grand-parental origin of alleles at markers and QTL. This kind of analysis is called pedigree-based linkage or cosegregation analysis. The accuracy of mapping a QTL by these methods depends on the number of recombinations or meioses within the pedigree. On the other hand, LD markers provide information to model both the mean and covariances at the linked QTL [7-11]. This LD information comes from the non-random association of allele states at markers and QTL. Before high density genotypes were available, LD between markers and QTL was created by crossing of two divergent lines. Given the high density genotypes that are currently available, markers that are in close proximity to QTL are expected to be in LD with the QTL. Thus LD or association mapping can now be undertaken in outbred populations without the need to create specialized crosses. These analyses that capture the information from LD markers for mapping and genetic evaluation are called population-based association or linkage disequilibrium (LD) analyses. Association analysis is expected to have higher accuracy than linkage analysis, but it is less robust to spurious association [12]. An analysis that combines the LD and CS information (LDCS analysis) has higher accuracy than LA analysis alone as well as greater robustness to spurious association than LD analysis alone [12,13]. Many methods have been proposed for the LDCS analysis. In some of these methods, phenotypes are modeled as a mixture distribution due to the segregation of the QTL. Analyses involving mixture distributions are computationally demanding [12,14-17]. Thus, other methods often model phenotypes as a normal distribution, where the mean and covariance matrix are computed conditional on marker information [3,13,18-25]. The method proposed in this paper belongs to the latter group.

An analysis that models the mean and covariances using LD markers was first proposed by Goddard [3] and was further developed by Wang et al. [18], when disequilibrium was entirely due to crossbreeding and the marker locus was assumed to be in equilibrium with the QTL in the parental breeds. Methodology to accommodate purebred populations with disequilibrium was considered by Fernando and Totir [23]. The parameters in their model included the mean and variance at the linked QTL for each marker haplotype in the founders [23], but did not specify the number of alleles at the QTL. Here, we consider a similar approach but following Fernando [22] and Johnson and Harris [26], we assume only two alleles at the linked QTL, which is also a common assumption in models where segregation of the QTL is explicitly modeled resulting in a mixture distribution for the phenotypes [7,12,14-17,27-29]. The parameters in this two-allele model include the gene frequency at the linked QTL for each marker haplotype in the founders and the additive effect of the QTL [22,26]. Harris [26] estimated these model parameters by restricted maximum likelihood [30]. One of the problems with this approach is that the number of gene frequencies to be estimated increases exponentially with the number of marker loci that are used to form haplotypes. The number of parameters to be estimated can be reduced by making assumptions about how LD is generated, which then provides a model for QTL gene frequencies for the different haplotypes [15]. In this paper a logit-normal prior probability density is considered for the QTL gene frequencies to accommodate relationships between QTL frequencies for different marker haplotypes.

In this paper we will first present the gene frequency model that combines linkage disequilibrium (LD) and cosegregation information, as first introduced by Fernando [22]. Then we will evaluate the performance of the model by determining the power of detecting a QTL within a given chromosomal region and precision for fine mapping of a QTL that has been detected to the given region, using high-density SNP genotypes by Bayesian analysis. To simplify the analysis we only consider data from unrelated individuals in a purebred population. Analysis of data from related individuals will be discussed in a subsequent paper. Results from the gene frequency models will be compared with those from QTL mapping by least squares regression analysis [31]. A method based on computing identical by descent (IBD) probabilities for the unobservable QTL given observable marker has also been used for LD mapping in livestock [32]. Previous studies, however, have shown that this IBD method and regression give comparable results (see Discussion) [31].

## Methods

### Gene Frequency Model

In the following we assume the QTL has been localized to a 1 cM segment of the genome, and it will be fine mapped within this region using biallelic single nucleotide polymorphism (SNP) markers. A single QTL with two alleles, *Q*_1 _and *Q*_2_, is assumed to be present on this segment of the genome, and this QTL will be referred to as the marked QTL (MQTL). All other QTL are assumed to be unlinked to the markers and are referred to as residual QTL (RQTL). All QTL are assumed to be additive.

Suppose genotypes at the MQTL were observed. Then, trait phenotypic values of individuals in a purebred population can be modeled as(1)

where ***y ***is the vector of trait phenotypic values, ***β ***is a vector of non-genetic fixed effects, ***μ ***is the QTL substitution effect, ***u ***is the vector of the sum of additive effects of all RQTL, ***e ***is a vector of residuals, and ***X***, ***Q ***and ***Z ***are known incidence matrices. Given data from *p *animals, the incidence matrix ***Q ***will have *p *rows and a single column, with row *i *of ***Q ***containing the number of *Q*_*2 *_alleles carried by animal *i*.

Now, for the situation considered here, the genotypes at the MQTL are not observed, and genotypes are available only at linked markers. Thus, ***Q ***is an unobservable random matrix. The usual mixed model methodology cannot accommodate models with unobservable incidence matrices. Thus we define(2)

where ***M ***denotes the observed genotypic information on markers, and E(***Q***|***M***) is the conditional expectation of ***Q ***given ***M***. Using the double-expectation theorem,(3)

so ***a ***in 2 is a random vector with null mean. Now, ***Qμ ***in 1 can be written as(4)

The level of LD between the marker and the QTL, which is usually quantified by the squared correlation (*r*^2^) between them, determines the ability to predict the allele at the QTL from the allele at the marker locus. Consider the following situations with different levels of LD. When the marker locus and the QTL are in LE (*r*^2 ^= 0), they are independent, thus the conditional mean E(***Q***|***M***) = *E*(***Q***) doesn't depend on marker information *M*. When the marker locus and the QTL are in LD (*r*^2 ^> 0), they are dependent, thus the conditional mean E(***Q***|***M***) depends on marker information *M*. When the marker locus and the QTL are in complete LD (*r*^2 ^= 1), they are perfectly correlated, thus the allele at the QTL can be predicted exactly from allele at the marker locus. These situations show that E(***Q***|***M***) depends on the LD between the markers and QTL. Thus by modeling the conditional mean of ***Qμ ***given marker information, E(***Q***|***M***)***μ***, captures the LD information for mapping the QTL. Although ***a ***has null mean, its covariance matrix depends on the marker information because of the cosegregation of the QTL and linked markers [2]. Thus modeling the covariance matrix of ***a ***given marker information, Cov(***a***|*M*), captures the cosegregation information for mapping QTL. In the following, we will denote the conditional expectation E(***Q***|***M***) by . Now the model for the trait phenotypic values can be written as(5)

Provided we can compute , all the incidence matrices in this model are known, and the mixed model equations for this model can be setup provided we can compute the inverse of the covariance matrix for each of the random vectors ***a ***and ***u ***. The covariance matrix for ***u ***is proportional to the additive relationship matrix ***A***. The inverse of the additive relationship matrix is sparse, and thus it can be computed efficiently [33]. On the other hand, the inverse of the covariance matrix for ***a ***is not sparse, and thus its computation is not efficient. However, ***Za ***can be written as ***Wv***, where

 and  are the additive effects of the maternal and paternal MQTL alleles of individual *i*, and ***W ***is a known incidence matrix relating *v *to *y*. It can be shown that the covariance matrix, **Σ**_*v *_, for *v *can be calculated using a simple recursive formula that also leads to an efficient algorithm to invert **Σ**_*v *_[23]. The model for trait phenotypic values now becomes(6)

When the marker locus is in equilibrium with the MQTL, the QTL and marker are independent. And as we will see in detail in the following section, each row of  will be a constant that is equal to twice the frequency of the QTL. Thus, ***Z******μ ***can be dropped from the model. In this situation, only cosegregation information will contribute to the analysis through the modeling of covariances among MQTL effects. When disequilibrium is complete and all marker genotypes are observed, E(***Q***|***M***) = ***Q***. Thus, in this situation, ***v ***is null, and after utilizing the disequilibrium information, cosegregation information does not contribute to the analysis. When disequilibrium is partial, E(***Q***|***M***) ≠ ***Q***, and ***v ***is not null. In this situation, disequilibrium information will contribute to the analysis through the model for the mean of MQTL effects, and cosegregation information will contribute to the analysis through the model for covariances between MQTL effects. These points are further clarified in the following sections, in which we will show how to compute  and the covariance matrix for ***v***.

### Mean of MQTL additive genetic values

Recall that the mean of MQTL effects is *μ*, where row *i *of ***Q ***has the number of *Q*_2 _alleles carried by animal *i*. Thus, the *i*^*th *^element of ***Q ***is the sum of two Bernoulli variables, *I*(*S*_*Q*_(*m*, *i*) = *Q*_2_|*M*), which is a variable indicating whether the maternal allele of *i *is a *Q*_2_, and *I*(*S*_*Q*_(*p*, *i*) = *Q*_2_|*M*), which is a variable indicating whether the paternal allele of *i *is a *Q*_2_. Now, ***Q***_*i *_has expected value:(7)

where

and *S*_*Q*_(*m*, *i*) is the maternal MQTL allele state and *S*_*Q*_(*p*, *i*) the paternal MQTL allele state of individual *i*. These probabilities depend on the location l of the QTL relative to the markers. Let *F*_*Q*_(*m*, *i*) = *H*_*j *_denote the event that the maternal MQTL allele of individual *i *originated in a founder with marker haplotype *H*_*j*_. Then, for a founder *i*,  can be written as(8)

where ***π***_*j *_is the conditional probability that a founder with marker haplotype *H*_*j *_has MQTL allele *Q*_2_. Similarly,  can be written as(9)

The ***π***_*j *_in 14 and 15 are the disequilibrium parameters. Thus, under equilibrium, when marker and QTL allele states are independent, the conditional probability of a *Q*_2 _allele on a founder haplotype does not depend on the marker alleles on that haplotype, i.e.,(10)

Because(11)

for all i,(12)

Similarly,(13)

Thus, from 7, 12 and 13, each row of  is a constant that is equal to twice the frequency of the QTL.

However, under disequilibrium, when marker allele states *S*_*A *_and QTL allele states *S*_*Q *_are not independent, the ***π***_*j *_are not all equal and it follows that  and  depend on the marker haplotypes and thus would be different for animals with different marker haplotypes. Thus vector  is not a vector of constants. This demonstrates that disequilibrium information contributes to modeling the mean of MQTL effects.

### Covariance of MQTL additive genetic values

Cosegregation information contributes to modeling the covariances of MQTL effects. The gametic value  is the product of a Bernoulli variable with probability parameter  and *μ*, thus the variance of  is(14)

and similarly, the variance of  is(15)

As it is shown by 12 and 13 that under equilibrium , thus the variance of MQTL gametic values does not depend on the marker genotypes. However, under disequilibrium,  and  thus the variance of MQTL gametic values depend on the marker genotypes. These variances contribute to the diagonal elements of the covariance matrix **Σ**_*v *_of the vector of gametic values. In this paper, we mainly focus on unrelated individuals, whose gametic values are uncorrelated, thus the off-diagonal elements of the covariance matrix are zero.

### Bayesian Inference

Bayesian methods will be used to make inferences on QTL effects and position under the statistical model described in the previous section. Given the high marker density being used in this paper, the QTL position is restricted to the midpoint between adjacent markers. In the Bayesian approach, prior knowledge about parameter values in a statistical model are quantified in terms of prior probabilities. Then, inferences about parameter values are based on posterior probabilities, which are obtained using Bayes theorem as(16)

where *f*(***y***|***θ***) is the conditional density of the data vector ***y ***given the vector of parameter values ***θ***, and f(***θ***) is the prior probability density of ***θ***.

In this paper we only consider a case with unrelated individuals, which allows RQTL effects to be merged with the residual effects of model (1). Cases with pedigree data will be covered in a subsequent paper. When individuals are unrelated, the gametic deviations of those individuals are also uncorrelated, thus cosegregation information can also be combined with the residual,(17)

This, however, results in the residual variances to be heterogeneous,(18)

Residual covariance matrix R* is diagonal with element  equal to  when individuals are unrelated. Now, the model simplifies to(19)

The parameters in model 19 are: *β*, , ***π***, *μ *and *l *because all other variables, such as  and , are functions of these parameters, as specified through equations 14 and 15. The size of the vector of conditional QTL probabilities of marker haplotypes, ***π ***is 2^*k *^when using haplotypes of *k *markers. In this study we only consider models where k is 1 or 2. When k is 1, the estimated QTL location was limited to the marker positions, and ***π ***is a vector of size 2 with elements corresponding to haplotypes 0 and 1 of the marker at the putative QTL location. When k is 2, the estimated QTL location was limited to the mid-points of adjacent markers, and ***π ***is a vector of size 4, with elements corresponding to haplotypes 00, 01, 10 and 11 of the two SNPs flanking the putative QTL location, with alleles denoted by 0 and 1.

The prior densities that were used for these parameters are described next. Following common practice, the priors given below were used for ***β ***and , which are parameters in the usual mixed linear model [34]. A flat prior was used for the fixed effects ***β***:(20)

The prior for  was taken to be scaled inverted chi-square distribution with degree of freedom *v*_*e *_and scale parameter ,(21)

The prior for ***π ***was taken to be logit-normal because this distribution can account for any correlations between elements of ***π***, which can range from 0 to 1. Thus the logit transformation of ***π***,(22)

was taken to be multivariate normal with null mean and covariance matrix **Σ**_*x*_. So the prior for ***π ***was written as(23)

where  is the Jacobian of the transformation. The covariance matrix **Σ**_*x *_accommodates covariances between elements of ***π***, which arises from the LD generating mechanism. In the following we, however, only consider the case where ***π***'s are uncorrelated, which means **Σ**_*x *_is diagonal.

The prior for the effect of the biallelic QTL, *μ*, was set to be normal with null mean and variance *σ*_*μ*_,(24)

The prior for location of the QTL, *l*, was taken to be a discrete uniform distribution. If there are L segments on the chromosome, the prior density for *l *was set to be(25)

It was further assumed that trait phenotypic values had a multivariate normal distribution given all location and dispersion parameters:(26)

Then the joint posterior density of parameters is(27)

Drawing inference directly from this posterior is impractical, so a Markov-chain was constructed for which 27 is the stationary distribution. Under certain conditions, samples drawn from such a chain can be used to make inferences on the parameters in 27 [34]. The most important conditions here are the existence of a unique stationary distribution and irreducibility of the chain [35]. As described below, a blocked Gibbs sampling strategy was used to construct a Markov Chain with stationary distribution 27. The sampler consisted of three blocks: fixed effect *β *was in the first block, ***π***, *μ *and *l *were in the second, and  was in the third. Parameters in each block were sampled from their full condition distributions, which are the conditional distributions of these parameters given parameters in other blocks and the phenotypic and marker data.

The conditional posterior distribution of fixed effect parameter *β *is(28)

where  is the solution to the mixed model equations, and ***C ***is the left hand side of mixed model equations. For each of the remaining parameter blocks, the full conditional posterior distribution does not have a standard form. Thus, Metropolis-Hasting algorithm was used. This requires a proposal distribution to draw the candidate samples from. The joint conditional posterior distribution of ***π***, *μ *and *l *is(29)

Rather than drawing samples from a proposal for ***π***, we draw samples from a proposal distribution of *x *and the sampled *x *is transformed to ***π***. The proposal for *x *was taken to be a multivariate normal distribution with mean equal to the value from the previous sample and variance **Σ**_*x*-*prop*_. Thus the proposal for *x *is(30)

Then, the proposal for ***π ***is(31)

where n is the size of vectors ***x ***and ***π***. The covariance matrix **Σ**_*x *_was set to ***I***, with  sufficiently small such that ***x ***will be sampled in the neighborhood of the previous sample. The proposal distribution of *μ *was taken to be normal with mean equal to the value from previous sample and variance  sufficiently small to ensure sampling in the neighborhood of the previous sample,(32)

The proposal for *l *was taken to be(33)

where L is the number of chromosome segments flanked by adjacent markers.

In the Metropolis-Hasting algorithm the candidate samples are accepted with probability [36]:(34)

where(35)

The full conditional posterior of  is(36)

Since R* is not equal to ***I***, the full conditional posterior of , does not have the form of the usual inverse chi-square distribution. Thus Metropolis-Hasting was used with a normal proposal(37)

to obtain candidate samples. The mean of this proposal distribution of  was set to the previously accepted value of , and variance  was set to a sufficiently small value to ensure sampling in the neighborhood of the previous sample. The candidate samples were also accepted with probability:(38)

where(39)

### Least squares analysis of regression method

Least squares regression to map a QTL using high-density SNP genotypes, as described by Grapes et al. [31], was used for comparison. The regression method on haplotypes is(40)

where *g*_*ij *_is the copy number of haploype j for individual i, and *b*_*j *_is the effect of haplotype j on phenotype. In this study we only consider models with 1 or 2 SNPs. For the 1-SNP regression method, there are two possible haplotypes 0 and 1, and the hypothesis *H*_0_: *b*_0 _= *b*_1 _vs *H*_a _: *b*_0 _≠ *b*_1 _was tested. For the 2-SNP regression method, there are four possible haplotypes 00, 01, 10 and 11, and the hypothesis *H*_0_: *b*_00 _= *b*_01 _= *b*_10 _= *b*_11 _vs *H*_*a *_: *b*_00 _≠ *b*_01 _or *b*_00 _≠ *b*_11 _or *b*_01 _≠ *b*_11 _was tested. This analysis was repeated for each SNP or SNP bracket. The estimated QTL location was at the SNP yielding the smallest p-value for the 1-SNP model, and at the midpoint of the SNP bracket yielding the smallest p-value for the 2-SNP model. When several locations had p-values numerically equal to zero, the middle location among those with zero p-values was chosen to be the QTL location.

### Simulation

Computer simulation was used to compare the power to detect and the precision to map QTL by Bayesian analysis using the gene frequency model (BGF) with least squares using the regression method (LSR). We simulated 2000 biallelic loci spaced either 0.01, 0.005 or 0.002 cM apart. Among these, every tenth locus was a QTL, and the remaining loci were markers. In the first generation, alleles were sampled independently from a Bernoulli distribution with probability 0.5. This generates a genome in Hardy-Weinberg and linkage equilibrium. LD was generated in this chromosomal segment by random mating with a mutation rate of 2.5 * 10^-5 ^and an effective population size of 500 for 1000 generations, followed by 50 generations of random mating with the population size reduced to 100. It has been estimated that the effective population size of livestock has decreased due to breed formation and artificial breeding [37]. The effective population sizes used in this simulation attempt to mimic this phenomenon. The initial allele frequencies of 0.5 and mutation rate of 2.5 * 10^-5 ^allow the population to approach mutation-drift equilibrium after the 1050 generations of random mating [38].

In the following, each set of 10 consecutive loci is referred to as a locus bin. Thus, there were 200 bins on the chromosomal segment that was simulated. In the final generation, out of each bin, the marker that had allele frequencies closest to 0.5 was selected. This generated markers spaced either 0.1, 0.05 or 0.02 cM apart. For the two-marker BGF and LSR analyses, marker haplotypes are assumed to be known. Out of the 200 QTLs, the QTL that had allele frequencies closest to 0.5 was identified. Markers for the analysis were chosen out of the selected markers from a chromosomal segment of 1 cM consisting of k consecutive locus bins. Thus, k was 10, 20, or 50 when marker spacing was 0.1, 0.05, or 0.02 cM. It is known that some methods of fine mapping are favored when the QTL is simulated at the center of the chromomsal segment [31]. Thus the identified QTL was simulated at a distance of 0.3 cM from the first marker locus in the segment. In addition to SNP density, the impact of sample size (500 or 1000) and of variance explained by the QTL (2% or 5% of the phenotypic variance) on power and precision were studied. Mean absolute error of estimates of QTL location was used as the statistic to quantify precision of QTL mapping. Power to detect the QTL was quantified as follows. For the regression method, the critical value for detecting a QTL was estimated by simulating data sets with no QTL and computing the upper 10% quantile F-value from 1500 replications of F-tests. Power was estimated by simulating data sets, each with one QTL, and calculating the percentage of F-values that were larger than the estimated critical value. For the gene frequency model, the estimate of QTL variance was used as the statistic to calculate power. The critical value for this test was estimated by simulating data sets with no QTL and computing the upper 10% quantile for the QTL variance from 1500 replications. Power was estimated by simulating data sets, each with one QTL, and calculating the percentage of estimates of QTL variance that are bigger than the estimated critical value. In this study the simulated true haplotypes were used for 2-SNP BGF and LSR.

## Results

### Power

For both 1 and 2-SNP BGF analyses, power to detect the QTL increased with sample size, QTL variance, and marker density (table 1). The 2-SNP BGF model seemed to have slightly higher power than the 1-SNP model.

**Table 1:** Power

QTL Var %	marker spacing (cM)	sample size	BGF1	BGF2	LSR1	LSR2
2	0.1	200	0.40	0.40	0.40	0.39
2	0.05	200	0.42	0.42	0.42	0.41
2	0.02	200	0.43	0.43	0.41	0.40
2	0.1	500	0.67	0.72	0.78	0.76
2	0.05	500	0.74	0.76	0.79	0.77
2	0.02	500	0.77	0.77	0.77	0.74
5	0.1	200	0.71	0.74	0.77	0.74
5	0.05	200	0.75	0.76	0.79	0.78
5	0.02	200	0.75	0.77	0.78	0.78
5	0.1	500	0.95	0.97	0.98	0.98
5	0.05	500	0.97	0.98	0.99	0.99
5	0.02	500	0.99	0.99	0.99	0.99

Power to detect a QTL using the gene frequency model (BGF) and the least squares regression model (LSR) with one marker (BGF1, LSR1) or two flanking markers (BGF2, LSR2) for different variances explained by the QTL (% of phenotypic variance), marker spacing, and sample size. For the regression method, the critical value for detecting a QTL was estimated by simulating data sets with no QTL and computing the upper 10% quantile F-value from 1500 replications of F-tests. Power was estimated by simulating 1500 data sets, each with one QTL, and calculating the percentage of F-values that were larger than the estimated critical value. For the gene frequency model, the estimate of QTL variance was used as the statistic to calculate power. The critical value for this test was estimated by simulating data sets with no QTL and computing the upper 10% quantile for the QTL variance from 1500 replications. Power was estimated by simulating 1500 data sets, each with one QTL, and calculating the percentage of estimates of QTL variance that are bigger than the estimated critical value

For both 1 and 2-SNP LSR analyses, power increased with sample size and QTL variance (table 1). Power also increased when marker spacing decreased from 0.1 to 0.05 cM but, in most cases, power decreased when marker spacing was further reduced to 0.02 cM. As described earlier, when markers were spaced 0.1, 0.05, or 0.02 cM apart, the number of markers or marker pairs in the chromosomal segment was 10, 20 or 50. The decrease of power when marker spacing dropped from 0.05 to 0.02 cM may be due to the increase in number of tests that were done to detect a significant QTL within the chromosomal segment. In all scenarios studied 1-SNP LSR had slightly greater power than 2-SNP LSR.

In most scenarios studied, both 1 and 2-SNP BGF had power close to those of LSR.

### Precision

The standard error of the mean absolute error of estimates of QTL location was about 0.003 for the 1 and 2-SNP BGF analyses. For almost all scenarios the 2-SNP BGF had almost the same precision as the 1-SNP BGF. For both analyses precision of estimates of QTL location increased with sample size and QTL variance (table 2). However, similar to the LSR, precision decreased when marker spacing decreased from 0.1 to 0.05 and 0.02 cM, except when sample size was 500 or the QTL explained 5% of phenotypic variance. The standard error of the mean absolute error for estimated QTL location of the 1 and 2-SNP LSR method was about 0.004 cM. For almost all scenarios the 2-SNP LSR had higher or same precision as 1-SNP LSR. In all scenarios, the 1 and 2-SNP BGF were consistently better in precision than the LSR, except for just one scenario when QTL explained 5% of phenotypic variance, marker spacing was 0.05 cM and sample size was 500, 1-SNP BGF and LSR had about the same precision. For both analyses, precision of mapping QTL increased with sample size and QTL variance (table 2). In most cases precision increased when marker spacing was reduced from 0.1 to 0.05 cM but remained unchanged when marker spacing was further reduced to 0.02 cM, except when sample size was 500 and the QTL explained 5% of phenotypic variance.

**Table 2:** Precision

QTL Var %	marker spacing (cM)	sample size	BGF1 (cM)	BGF2 (cM)	LSR1 (cM)	LSR2 (cM)
2	0.1	200	0.18^*a*^	0.17^*b*^	0.23^*c*^	0.21^*d*^
2	0.05	200	0.19^*a*^	0.19^*b*^	0.23^*c*^	0.23^*c*^
2	0.02	200	0.21^*a*^	0.21^*b*^	0.25^*c*^	0.23^*d*^
2	0.1	500	0.15^*a*^	0.14^*a*^	0.19^*b*^	0.18^*b*^
2	0.05	500	0.15^*a*^	0.15^*b*^	0.17^*c*^	0.17^*c*^
2	0.02	500	0.16^*a*^	0.16^*b*^	0.18^*c*^	0.18^*c*^
5	0.1	200	0.15^*a*^	0.14^*b*^	0.19^*c*^	0.18^*c*^
5	0.05	200	0.16^*ab*^	0.15^*b*^	0.17^*ab*^	0.17^*ac*^
5	0.02	200	0.17^*a*^	0.16^*b*^	0.18^*c*^	0.17^*c*^
5	0.1	500	0.14^*a*^	0.14^*bc*^	0.16^*a*^	0.15^*ac*^
5	0.05	500	0.12^*a*^	0.11^*a*^	0.12^*bd*^	0.12^*cd*^
5	0.02	500	0.11^*a*^	0.10^*b*^	0.12^*cd*^	0.12^*ad*^

Precision to map a QTL using the gene frequency model (BGF) and the least squares regression model (LSR) with one marker (BGF1, LSR1) or two flanking markers (BGF2, LSR2) for different variances explained by the QTL (% of phenotypic variance), marker spacing, and sample size. Mean absolute error of estimates of QTL location was used as the statistic to quantify precision of QTL mapping. Paired t-tests were done to test whether the pairwise differences between the BGF1, BGF2, LSR1 and LSR2 are significant or not for all twelve different scenarios. The results are based on 1500 simulating data sets. ^*a, b, c, d*^Within a row, means without a common superscript differ (*P *< 0.05).

**Figure 1:**
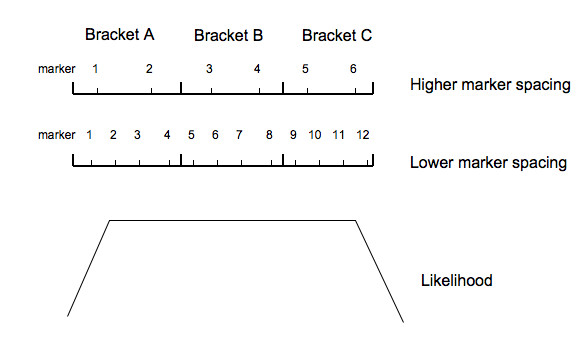
**Likelihood plateau under high and low marker spacing**. When there is not sufficient information, the likelihood will not peak at the location of the QTL, but may have a plateau centered at the QTL location. With the higher marker spacing, four markers are on the plateau of the likelihood, of which two are inside bracket B. Thus the QTL has probability 0.5 to be mapped inside bracket B. With lower marker spacing, ten markers are on the plateau, of which six are outside and four are inside bracket B. Thus the QTL has a higher probability to be mapped outside than inside bracket B, which results in lower precision. However, when there is sufficient information due to a larger number of observations or higher QTL variance, the likelihood will be more peaked. Thus there is less probability that the QTL will be mapped outside of bracket B, resulting in a higher precision with a decrease in marker spacing.

The fact that precision doesn't increase with the decrease of marker spacing for both BGF and LSR analysis shows that without enough information, higher marker density does not necessarily result in higher precision for mapping. If sample size or QTL variance was sufficiently high, precision increased with the increase of marker spacing. The reason for this is that, when there is not sufficient information, the likelihood will not peak at the location of the QTL, but may have a plateau centered at the QTL location, as shown in Figure 1. With the higher marker spacing, four markers are on the plateau of the likelihood, of which two are inside bracket B. Thus the QTL has probability 0.5 to be mapped inside bracket B. With lower marker spacing, ten markers are on the plateau, of which six are outside and four are inside bracket B. Thus the QTL has a higher probability to be mapped outside than inside bracket B, which results in lower precision. However, when there is sufficient information due to a larger number of observations or higher QTL variance, the likelihood will be more peaked. Thus there is less probability that the QTL will be mapped outside of bracket B, resulting in a higher precision with a decrease in marker spacing. In all scenarios studied, both 1 and 2-SNP BGF had precision higher than LSR.

## Discussions

In this study, we have presented a gene frequency model that combines LD and cosegregation information for use in fine mapping of QTL. In this method LD information is captured by modeling the conditional mean of the QTL given marker information, and cosegregation information is captured by modeling the covariance matrix of the QTL given marker information. This model can accomodate situations when there is no LD and only cosegregation information as well as only LD and no cosegregation information. It should be noted that using 13 leads to an approximation of the covariance matrix and its inverse when marker data are not complete. Complete marker data in this situation are the ordered genotypes at the marker locus. Wang et al. [39] gave a recursive formula that gives exact results with unordered genotypes at a single locus. The advantage of using 13 to compute **Σ**_*v*_, however, is that this leads to an efficient algorithm to invert this covariance matrix [23], and without such an algorithm, genetic evaluation with large pedigrees may not be possible. Recently, however, Thallman et al. [40] developed a recursive formula that gives exact results with missing genotypes for a pedigree with loops. Implementation of this algorithm is, however, beyond the scope of this paper.

Least squares regression, which is easy to implement and computationally efficient, was used to compare to the gene frequency model in power and precision of QTL mapping. Besides the regression method, an identity by descent (IBD) method has been proposed for QTL mapping by Meuwissen and Goddard [32]. This method is based on computing IBD probabilities between QTL alleles on haplotypes of relatives given the similarity between marker alleles on these haplotypes. An algorithm was developed to approximate the probability that the alleles at the QTL are IBD given the number of marker alleles that are consecutively identical in state to the left and right of the QTL [41].

Grapes et al. [31] studied the precision of QTL mapping using the IBD and regression methods. When markers were spaced 1, 0.5, or 0.25 cM apart, the IBD method with 10 markers had higher precision in mapping than regression with 10 markers. In a subsequent study, Grapes et al. [42] showed that the IBD method with 4-6 markers led to higher precision than with 10 markers. In both these studies, markers were used in the analysis even if they were fixed after 100 generations of random mating. Using only markers that are segregating after 100 generations of random mating, Zhao et al. [43] studied power and precision of the regression and IBD methods under scenarios with different marker spacing and percentage of phenotypic variance explained by QTL. Using four or six markers gave best result for the IBD method for both power to detect and precision to map a QTL, but regression with 1 SNP had even higher precision, except in one scenario where the IBD method was better. The IBD method had higher power than regression, except for two scenarios with higher marker density, where regression had the same or higher power than the IBD method. Because results from regression were close to or better than those from the IBD method, regression was used in this study to compare with the gene frequency model in power and precision of QTL mapping. Calus et al. [44] compared the accuracy of predicting breeding values in genomic selection for regression with 1 marker haplotypes, 2 markers haplotypes, IBD with 2 markers haplotypes and IBD with 10 markers haplotypes. The marker density simulated in their study was 2343, 1166.4, 463.9 232.1 or 119 polymorphic markers across 3 M genome, and heritability of the trait was 10 or 50%. Thus marker densities in their study were much lower than in this paper. At lower marker densities, IBD with 10 markers always had the highest accuracy of estimated breeding values, and regression with one marker had the lowest accuracy. As marker density increased, the difference in accuracies decreased. However, at the highest marker density, when heritability was 10%, regression with 1 marker had the highest accuracy. Thus, since in this paper, marker densitites were much higher, it is expected that the difference between the performance of regression and IBD method would be negligible.

The least squares regression method with one SNP had slightly higher power than with two SNPs for most of the scenarios studied. These results on LSR are consistent with those from Zhao et al. [43], who found that LSR with one SNP gave similar or higher power than with two SNPs, especially with high marker density. Unlike LSR, the gene frequency model with two SNPs had similar or slightly higher power than the BGF with one SNP. Both 1- and 2-SNP BGF Models had power close to the 1- or 2-SNP regression methods.

LSR with two SNPs had similar or slightly higher precision of mapping QTL than with one SNP. Grapes et al. [31] found that regression with one SNP had better precision than two SNPs, except for one scenario where they had the same precision. In their study, 10 or 20 evenly spaced biallelic markers were simulated within a 2.25-9 cM region in the base population, and all markers were used for mapping after 100 generations of random mating. This would result in some markers that are fixed, which wouldn't contribute to the analysis. However, in practice, uninformative SNPs will not be used in the analysis. In the present study and in that by Zhao et al. [43], only markers that were segregating were chosen for analysis. Zhao et al. [43] found that LSR with one SNP had higher precision than LSR with two SNPs. This result is not in agreement with our results, and may be due to the higher marker densities in our study, with 11, 21, 51 markers in a 1 cM region compared to 6, 10, 20 markers in an 11 cM region in the study by Zhao et al [43]. With the higher marker density, LD would be stronger, thus regression on one or two SNPs would not be much different, compared to lower marker density. BGF with two SNPs gave similar or higher precision than with one SNP. Both 1- or 2-SNP BGF models had higher precision than the 1- or 2-SNP LSR models. When marker density is high, sample size and QTL variance are large, BGF and LSR models converge in both power and precision. In the study by Calus et al. [44], difference in the accuracy of estimated breeding values between IBD and regression method was lowest at the highest marker density.

The essential difference between the BGF and regression model is the heterogeneous variance of the BGF residuals, which can be seen from (18). However, when ***π ***is 0 or 1, as can be seen from (14) and (15)  and  will also be 0 or 1 when haplotypes are known, which is always true for one-marker haplotypes and was also assumed for two-marker haplotypes in this paper. In this case, there is no heterogeneity of BGF residuals and the two methods will have the same performance. When all elements in ***π ***are 0 or 1, it implies complete LD between marker and the QTL. However, analyses of high-density SNP data in livestock have shown that LD between adjacent marker loci is not complete [45-48].

One of the advantages of the gene frequency model is that it can be used to combine linkage disequilibrium and cosegregation information for QTL mapping. However, here its performance was studied only for the simple case with unrelated founder individuals, where only LD contributes to the analysis. Thus in this case, the primary difference between the two models is that in the gene frequency model residual variances are heterogeneous (see equation 18), whereas in the regression model residual variances are assumed homogeneous.

Another difference between these two models is the assumption of biallelic QTL in the gene frequency model. This assumption is often made in Maximum Likelihood and Bayesian QTL mapping methods for mixture models because it is a good approximation, although the number of QTL alleles is unknown and difficult to infer in outbred populations [49]. Biallelic QTL methods have been shown to successfully detect linkage for multiallelic QTLs [50-53]. A comparison between the performance of mutiallelic and biallelic analyses under multiallelic modes of inheritance using the package Loki and the multiallelic version of Loki (maLoki) was done by Rosenthal et al. [54] using both simulated and real data. For simulated data a four-generation pedigree with 98 individuals was simulated to detect the linkage of a six-allele trait gene. Although the multiallelic analysis had better mixing and convergence than the biallelic analysis, the biallelic analysis was better at detecting linkage, and it had a lower bias in estimating the QTL position and the number of QTL. For real data 8 pedigrees with 216 individuals were used to detect linkage of APOC3 gene with QTL for high-density lipoprotein (HDL). Both biallelic and multiallelelic analysis had good mixing. Both the biallelic and multiallelic analyses fitted one or more QTL with probability almost one, while the probability of fitting two or more QTL was .27 for multiallelic analysis and .61 for biallelic analysis. However, the parameter estimates for the larger QTL were very similar. And their estimates are close or the same in posterior mean, standard deviation and range of total number of QTLs, and in posterior mean of QTL. Due to the good performance of biallelic analysis and increased computational cost of multiallelic analysis, biallelic analysis can be a good approximation that computationally easier and more feasible. The BGF model, however, can be extended to accommodate QTL with any specified or even unspecified number of alleles. If the number of alleles is not specified, it can be made to be an unknown parameter in the model with some prior distribution. But this will lead to more parameters that need to be estimated, thus will affect the power and precision of the analysis.

The 2-SNP BGF performed slightly better with regard to power and precision of QTL mapping than the 1-SNP BGF. It should be noted that the BGF method requires knowing the haplotypes for founder individuals. Haplotypes at a single locus can be determined from the genotype of the individual at this locus, the haplotypes for 2 or more loci cannot be inferred from the genotypes of the individual at these loci. Thus for 2-SNP BGF in practice haplotypes probabilities have to be calculated using genotypes of the individual, its ancestors and descendents. In this study the simulated true haplotypes were used for 2-SNP BGF. Thus, in practice, when haplotype probabilities are used, the slight advantage of 2-SNP BGF may not persist.

## Competing interests

The authors declare that they have no competing interests.

## Authors' contributions

The gene frequency model was developed by RLF and HG. WH and RLF developed the Bayesian analysis for the gene frequency model. WH programmed the algorithm in C++. WH, RLF and JCMD contributed to the design and implementation of the simulation to study the performance of the method. The manuscript was drafted by WH and RLF with contributions for revision by JCMD and HG. All authors read and approved the final manuscript.
